# Transmission ratio distortion of mutations in the master regulator of centriole biogenesis PLK4

**DOI:** 10.1007/s00439-022-02461-w

**Published:** 2022-05-10

**Authors:** Heidemarie Neitzel, Raymonda Varon, Sana Chughtai, Josephine Dartsch, Véronique Dutrannoy-Tönsing, Peter Nürnberg, Gudrun Nürnberg, Michal Schweiger, Martin Digweed, Gabriele Hildebrand, Karl Hackmann, Manuel Holtgrewe, Nanette Sarioglu, Bernt Schulze, Denise Horn, Karl Sperling

**Affiliations:** 1 Institute of Medical and Human Genetics, Charité-Universitätsmedizin Berlin, Berlin, Germany; 2 Cologne Center for Genomics, University of Cologne, University Hospital Cologne, Cologne, Germany; 3 Center for Molecular Medicine Cologne, Laboratory for Epigenetics and Tumour Genetics, University of Cologne, Cologne, Germany; 4 Institut Fuer Klinische Genetik, Medizinische Fakultaet Carl Gustav Carus, Technische Universitaet Dresden, Dresden, Germany; 5 Berlin Institute of Health (BIH), Core Unit Bioinformatics, Charité-Universitätsmedizin Berlin, Berlin, Germany; 6 Institute of Pathology, Charité-Universitätsmedizin Berlin, Berlin, Germany; 7 The Genetics Clinic, Hannover, Germany

## Abstract

The evolutionary conserved Polo-like kinase 4 (PLK4) is essential for centriole duplication, spindle assembly, and de novo centriole formation. In man, homozygous mutations in *PLK4* lead to primary microcephaly, altered *PLK4* expression is associated with aneuploidy in human embryos. Here, we report on a consanguineous four-generation family with 8 affected individuals compound heterozygous for a novel missense variant, c.881 T > G, and a deletion of the *PLK4* gene. The clinical phenotype of the adult patients is mild compared to individuals with previously described *PLK4* mutations. One individual was homozygous for the variant c.881G and phenotypically unaffected. The deletion was inherited by 14 of 16 offspring and thus exhibits transmission ratio distortion (TRD). Moreover, based on the already published families with *PLK4* mutations, it could be shown that due to the preferential transmission of the mutant alleles, the number of affected offspring is significantly increased. It is assumed that reduced expression of *PLK4* decreases the intrinsically high error rate of the first cell divisions after fertilization, increases the number of viable embryos and thus leads to preferential transmission of the deleted/mutated alleles.

**Supplementary Information:**

The online version contains supplementary material available at 10.1007/s00439-022-02461-w.

## Introduction

Primary autosomal recessive microcephaly (MCPH) is a rare fetal neurodegenerative disorder. At birth the head circumference is generally under the 3rd percentile and the body length slightly reduced. About 30 genes underlying MCPH, including Seckel syndrome, have been identified (Jayaraman et al. 2018; Naveed et al. 2018; Siskos et al. 2021). Interestingly, almost all *MCPH* genes are involved in the regulation of mitosis, preferentially by affecting the function of the centrosome, the major microtubules organizing centre.

Typically, each centrosome comprises two centrioles. During mammalian oogenesis the centrioles are destroyed and consequently the centrosomes of oocytes do not contain centrioles. In man, but not in mice, the sperm contributes a typical and a novel atypical centriole at fertilization, both functioning in the zygote and facilitating all further development (Fishman et al. 2018). Dysfunctional and supernumerary centrosomes promote mitotic instability in meiotic oocytes and at early embryogenesis which is considered the main reason for the high loss of human preimplantation embryos (Schatten and Sun 2011). Moreover, loss of centrosome numerical integrity is also involved in tumorigenesis due to its promotion of genome instability (Cappello et al. 2014).

The Polo-like kinase 4 (PLK4) is essential for centriole duplication (Sonnen et al. 2013; Yamamoto and Kitagawa 2021), for spindle assembly in the absence of centrioles (Coelho et al. 2013), and for de novo centriole formation (Eckerdt et al. 2011). Homozygous mutations in *PLK4* lead to primary microcephaly, combined with growth retardation and retinopathy (Martin et al. 2014; Shaheen et al. 2014; Tsutsumi et al. 2016; Dincer et al. 2017; Martin-Rivada et al. 2020), while heterozygous mutations might be associated with azoospermia (Miyamoto et al. 2016). This, however, applies to one case only, has not been validated in an independent study (Cioppi et al. 2021), and was not observed in the other heterozygote PLK4 probands published so far. Moreover, altered *PLK4* expression combined with chromosome instability is common in mouse and human cancers (Rosario et al. 2010; Ko et al. 2005) and associated with aneuploidy in human embryos (McCoy et al. 2015a, b, 2018). In mice, null mutations in *Plk4* are embryonic lethal (Hudson et al. 2001).

Here, we report on a consanguineous four-generation family with 8 affected individuals compound heterozygous for a novel missense variant and a deletion of the *PLK4* gene. The deletion was inherited by 14 of 16 offspring, both through oogenesis and spermatogenesis and thus exhibits transmission ratio distortion (TRD).

## Materials and methods

Peripheral blood was obtained from all individuals of generation III from II.1, II.3, and II.4, as well as from IV.1, IV.3, and IV.8, after informed consent. Tissues were available from the aborted fetuses IV.2–IV.6, chorionic villi from IV.7, and fibroblasts from IV.6. Lymphoblastoid cell lines were established from III.1 to III.6 (Neitzel 1986). Genomic DNA was extracted according to standard procedures.

### Linkage analysis

Genome-wide linkage was performed using the Affymetrix GeneChip® Human Mapping 10 K and 250 K Sty Arrays (Affymetrix, Santa Clara, CA). Relationship errors were evaluated with the help of the program Graphical Relationship Representation (Abecasis et al. 2001). The program PedCheck was applied to detect Mendelian errors (O´Connell and Weeks 1998), Non-Mendelian errors were identified by the program MERLIN (Abecasis et al. 2002). Linkage analysis was performed assuming autosomal recessive inheritance, full penetrance and a disease gene frequency of 0.0001. Multipoint LOD scores were calculated using the program ALLEGRO (Gudbjartsson et al. 2000). Haplotypes were reconstructed with ALLEGRO and presented graphically with HaploPainter (Thiele and Nuernberg 2005). All data handling was performed using the graphical user interface ALOHOMORA (Ruschendorf and Nuernberg 2005).

### Whole-exome sequencing

Whole-exome sequencing using the ABI SOLiD platform was performed following enrichment of exonic sequences using Agilent's SureSelect whole-exome enrichment. Called variants were filtered to exclude variants not found in all affected persons as well as common variants identified in the dbSNP130 or HapMap databases and characterized by PolyPhen2 (Adzhubei et al. 2010) and mutation taster (Schwarz et al. 2010).

### Whole-genome sequencing (WGS) and bioinformatics

Genomic DNA was obtained from blood samples and libraries were constructed according to the TruSeq PCR free or nano protocol. After passing quality control, DNA fragments with an insert size between 340 and 570 bp were subjected to sequencing on a HiSeq X Ten platform with 150 bp paired-end protocol until minimum mean coverage of 30 × per sample was reached. The sequence reads were aligned using BWA–MEM, duplicates were masked using Samblaster, and the resulting SAM files were converted to BAM and sorted using Samtools (Li et al. 2009; Faust and Hall 2014). SNVs and small indels were called using the UnifiedGenotyper (after realignment) and the HaplotypeCaller tools from GATK with default settings (DePristo et al. 2011). SNVs and small indels were filtered to a high-confidence de novo candidate call sets in a fashion similar to the one described by Besenbacher et al. (2015). First, variants were filtered to those showing a de novo genotype pattern: heterozygous alternative in index and homozygous reference in the parents. Second, variants were excluded falling into the UCSC “simpleRepeat” or “repeat_masker” track and also those having a value below 1 in the UCSC track “wgEncodeCrgMapabilityAlign36mer”. Third, a minimum genotype quality of 50 was required, a coverage between 10 and 120 reads, and a fraction of alternative reads at the variant site between 0.2 and 0.8. Finally, we applied filters similar to Wong et al. to remove potential false positives caused by mapping errors (Wong et al. 2016).

### Array CGH (aCGH)

Molecular karyotyping was performed using the Human Genome CGH Microarray Kit 244A (Agilent, Santa Clara CA, USA). Scanning of the hybridized array was carried out on an Agilent microarray scanner. Raw data were processed by the Feature Extraction 9.5.3.1 (Agilent) software. Deleted or amplified regions were determined by the CGH Analytics 3.5 (Agilent) program.

### Microsatellite analysis

Linkage analysis based on semi-automated genotyping was carried out with microsatellite markers on DNA samples of all individuals of generation III and IV, including II-1 and II-3. Microsatellite markers and their distances were from the Marshfield linkage map. 12 microsatellites, flanking the *PLK4* gene on chromosome 4q28 were analyzed. Moreover, more than 100 markers from candidate genes or gene regions on chromosomes 1, 3, 6, 12, 14, 18, and 19 were tested in a subset of individuals. Microsatellites were amplified by touchdown polymerase chain reaction (PCR) using fluorescently labeled primers as described elsewhere (Vanita et al. 2006). Data were collected and analyzed by GENESCAN version 3.1.2 (Applied Biosystems, Foster City, CA), and genotyping was done using GENOTYPER 2.5.1 software (Applied Biosystems, Foster City, CA). Recombination frequencies (θ) were considered equal between males and females. Two-point linkage analysis was performed by MLINK from the LINKAGE program package (Lathrop et al. 1984), and multipoint analysis was undertaken using GENEHUNTER (Kruglyak et al. 1996).

### Mutation screening

Mutation screening in more than 60 candidate genes was performed by bidirectional sequencing of PCR products. The PCR primers were designed using UCSC data base (http://genome.ucsc.edu/) for all exons and intron–exon boundaries. PCR products were sequenced by BigDye Terminator method on an ABI 3730 sequencer.

### cDNA analysis of the PLK4 gene

RNA was extracted from lymphocytes using the RNeasyr Mini Kit (Quiagen, Hilden, Germany). Total RNA was reversed transcribed using the RevertAid tm First Strand cdna SYNTHESIS Kit (Fermentas, St. Leon). The RT-PCR products were used for PLK4-specific PCR amplification.

### Immunohistochemistry

Fibroblast lysates from a control and from the affected fetus IV.6 were resolved on a 4–12% Bis–Tris polyacrylamide gradient gel (NuPage, Invitrogen). Proteins were transferred to an Invitrolon PVDF membrane (Invitrogen) which was then blocked for at least 1 h in 10% non-fat milk in Tris-buffered saline, pH 7.6, with 0.1% Tween-20 (TBS-T). Incubation with primary and secondary antibodies was performed in 5% non-fat milk in TBS-T. All washing steps were carried out using TBS-T. Immunoblots were probed with a murine monoclonal antibody directed against amino acids 1–110 of human PLK4 (Abcam, ab56752) and a murine monoclonal antibody directed against rabbit muscle GAPDH (Thermo Fisher Scientific, AM4300). Primary antibodies were detected with horseradish peroxidase-conjugated goat anti-mouse IgG (GE Healthcare, NA931V). Chemiluminescence was developed using Western Lightning (PerkinElmer Life Sciences, Boston, MA, USA).

## Results and discussion

**Fig. 1:**
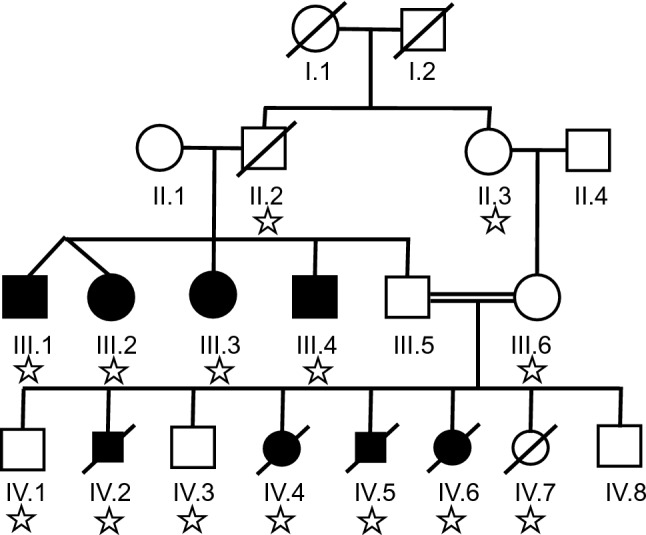
Pedigree of the family with autosomal recessive primary microcephaly in generation III and IV. The *PLK4* deletion (Star symbol) was inherited to 14 of the 16 offspring of generation I, both through spermatogenesis (II.2) and oogenesis (II.3, III.6). The complete PLK4 constitution including the novel variant is depicted in Fig. 3

The analysis is based on a four-generation family. The unaffected male of the core family (III.5 in Fig. 1) has four sibs all affected with microcephaly, intellectual disability, and the characteristic facial aspect of autosomal recessive primary microcephaly (sloping forehead, micrognathia). Two of them also exhibit short stature, two were in the lower normal range. All attended a school for mentally handicapped and were able to speak in sentences (Table 1).

**Table 1:** Clinical data of the four affected individuals in generation III

Individual	III.1	III.2	III.3	III.4
Age at clinical investigation (years)	30	30	26	24
Sex	Male	Female	Female	Male
Low birth weight	nd	nd	nd	– (3000 g)
Body length (cm)	144 ( – 5.3 SD)	135 ( – 5 SD)	153 ( – 2.3 SD)	164 ( – 2.3 SD)
Weight (kg)	50.9	34.5	62.5	60.0
Head circumference (cm)	46.3 ( – 7.0 SD)	43.5 ( – 12.6 SD)	47.5 ( – 7.4 SD)	48.8 ( – 5.3 SD)
Craniofacial dysmorphism	Sloping forehead, micrognathia	Sloping forehead, micrognathia	Sloping forehead, micrognathia	Sloping forehead, micrognathia
Intellectual disability	Moderate	Moderate	Moderate	Moderate

*nd* no data

The unaffected III.5 has together with his cousin III.6 eight offspring, among them four fetuses with severe primary microcephaly diagnosed in the first trimester of pregnancy by ultrasound. All four affected fetuses presented with microcephaly and retrognathia already present at 15 gestational weeks. A detailed autopsy of the fetal brain of IV.2 showed an extensive migration impairment of the cerebral hemispheres, a developmental disorder of the hippocampal formation and hypoplasia of the cerebellar hemispheres. The pregnancies with the affected fetuses were terminated on maternal psychological grounds.

The common grandparents (I.1, I.2) of III.5 and III.6 were unrelated; however, their ancestors did come from the same small village. The mother (II.1) of the four affected sibs (III.1–III.4) is clinically inconspicuous and her head circumference at 56.5 cm is in the normal range. All patients and their unaffected relatives were examined by a clinical geneticist (Table 1).

Since III.5 and III.6 are first cousins, we expected that the affected offspring would be homozygous for the same recessive variant in a candidate gene involved in mitosis. Neither Sanger nor whole exome sequencing (WES) indicated a homozygous variant of clinical significance in any of the relevant candidate genes (Suppl. Table 1). Genome wide SNP linkage analysis (10 K array) of the affected fetuses in generation IV revealed a compound heterozygous region of 29.7 Mb on chromosome 4 from 109,289,155 (rs141066) to 139,029,890 (rs1112918) harboring the *PLK4* gene. The affected individuals of generation III shared a compound heterozygous region of 40.7 Mb on chromosome 4, which is, however, localized distally of *PLK4*, from 131,116,040 (rs17050775) to 171,779,140 (rs10017619). Thus, at the position of *PLK4* the compound heterozygous regions of generation III and IV do not overlap, indicating either different candidate genes segregating in the family or a more complex mechanism explaining the inheritance of microcephaly in this family. Our attempt to map the region around PLK4 using flanking microsatellite markers was inconclusive due to crossover events in III.4, III.5 and III.6 (Suppl. Figure 1).

**Fig. 2:**
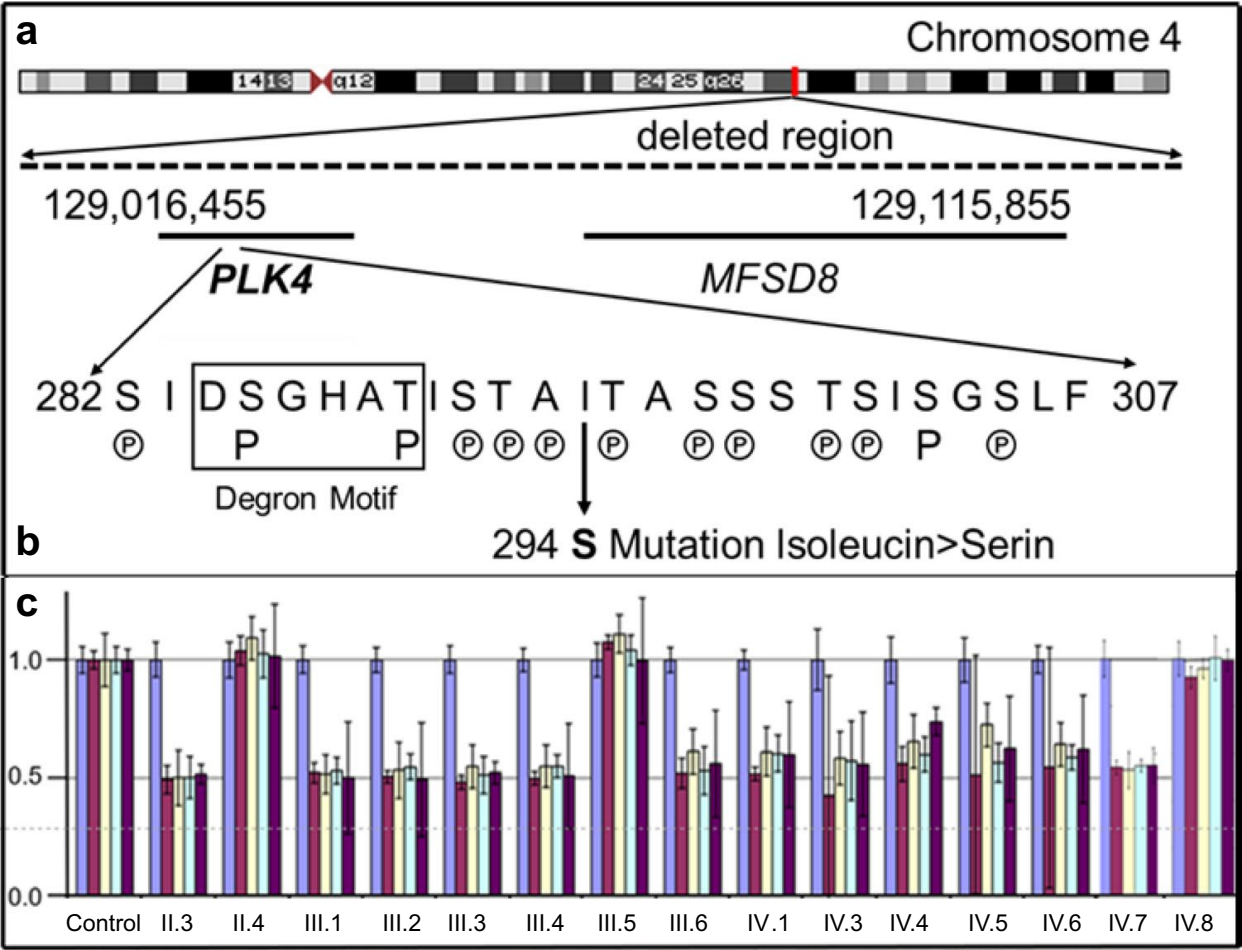
**a** Deleted region of chromosome 4 identified by array-CGH. **b** Novel missense variant (c.811 T > G) in *PLK4* near the Degron motif resulting in the replacement of isoleucine by serine (p.294Ile > Ser). The known (P) and putative autophosphorylation sites ℗, which regulate the degradation of the protein, are indicated (after www.phosphosite.org/). Note, that the mutation creates a potential new autophosphorylation site. **c** qPCR analysis of exons 4, 5.1, 5.2 and 6 of *PLK4* (brown–purple) and as a control exon 2 of the *Cystic fibrosis transmembrane conductance regulator (CFTR)* gene (blue)

Therefore, we performed a high-resolution array-CGH (aCGH) with DNA from three affected probands, one from generation III (III.2) and two from generation IV (IV.5, IV.6). The aCGH demonstrated a deletion spanning the complete *PLK4* gene, the complete *MFSD8* gene and exon 1 of the *ABHD18* gene (Fig. 2a). Based on WGS the size of the deletion is 115,948 bp (129,002,909 to 129,118,857) and the existing *MSFD8* and *ABDH18* genes are unaffected.

The *MFSD8* gene encodes a ubiquitous integral membrane protein that contains a transporter domain and a major facilitator superfamily (MFS) domain. Mutations in this gene are correlated with autosomal recessive neuronal ceroid lipofuscinoses and macular dystrophy with central cone involvement (https://www.deciphergenomics.org/gene/MFSD8/overview/clinical-info). The ABHD18 (Abhydrolase Domain Containing 18) gene is predicted to be located in the extracellular region. So far, no phenotypes are associated with sequence variants (https://www.deciphergenomics.org/gene/ABHD18/overview/clinical-info).

In Western blots of the affected fetus IV.6, we observed a single PLK4 band and a reduction in the amount of protein by 50% compared to the control (Suppl. Figure 2b).

To confirm the deletion in other family members, qPCR was performed indicating that all four affected members of generation III carry the deletion inherited on the paternal haplotype.

The mother III.6 and grandmother II.3 were carriers of the deletion but unaffected and III.6 transmitted the deletion to seven of her eight offspring, four of whom were affected by early prenatal onset of microcephaly (Figs. 1, 2). Thus, the segregation pattern in this family was indicative for recessive inheritance.

**Fig. 3:**
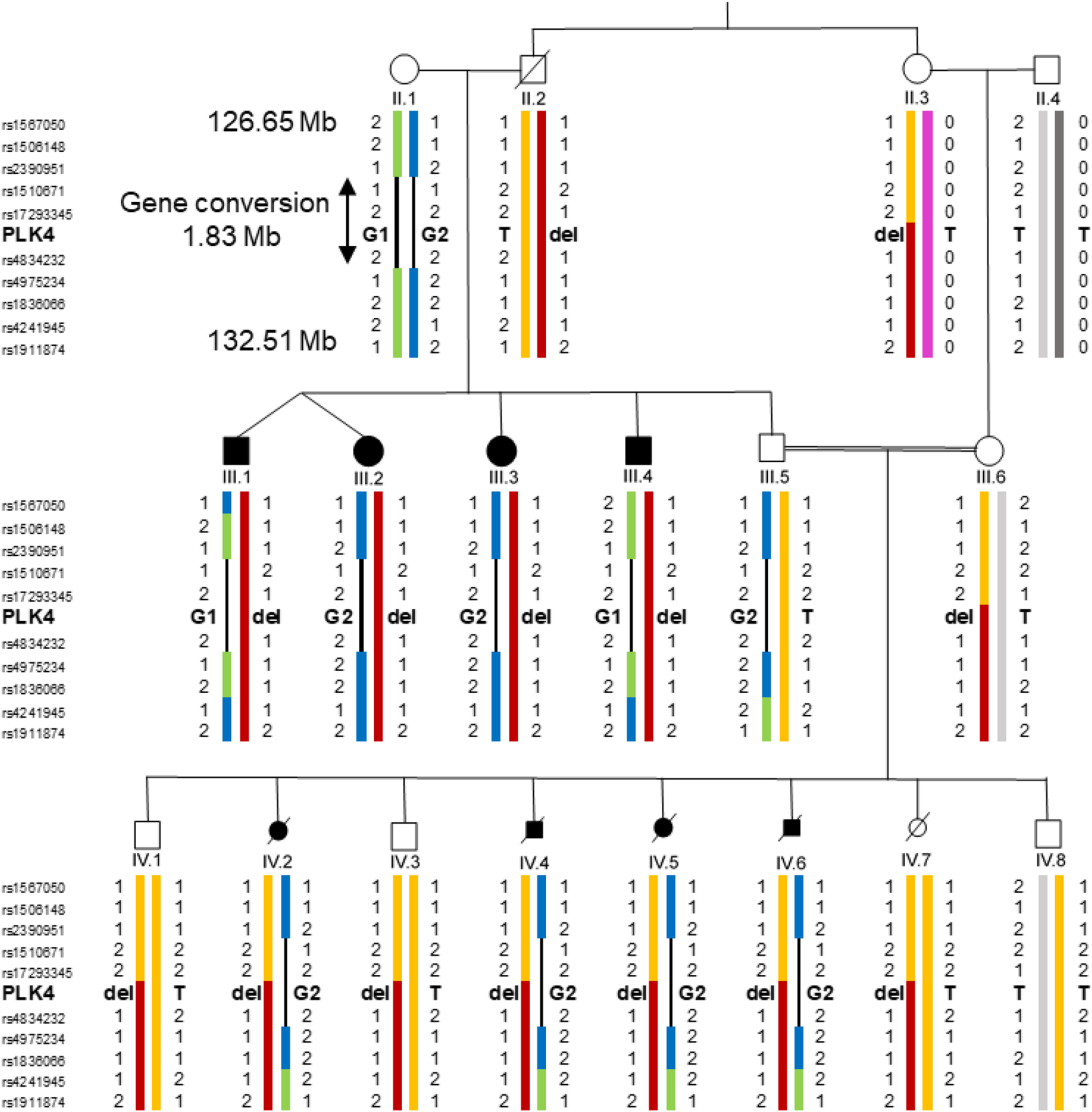
Reconstruction of haplotypes based on manual analysis of SNPs and microsatellites (Suppl. Figure 1) flanking the *PLK4* gene and the region of gene conversion. The color changes point to the sites of crossovers. green—grandmother (II.1) haplotype G1 with the mutated *PLK4* allele c.881G, blue—grandmother haplotype G2 with the mutated *PLK4* allele, red—grandfather (II.2) haplotype with the deletion, yellow—grandfather haplotype with the wild-type *PLK4* allele c.881 T, purple—grandmother (II.3) haplotype with the wild-type *PLK4* allele, light and dark gray—grandfather (II.4) haplotypes with the wild-type *PLK4* alleles

Subsequent sequencing of all *PLK4* exons and the exon–intron boundaries demonstrated that all affected probands with the deletion of *PLK4* carry a *PLK4* variant c.881G (Suppl. Figure 2a). The variant has not been reported in any public database. All unaffected individuals with the deletion have the *PLK4* wildtype allele c.881 T (II.3, III.6, IV.1, IV.3). The healthy father of generation IV (III.5) is heterozygous c.881 T/G, while the unaffected grandmother, II.1, is homozygous for the variant c.881G (Fig. 3). The base pair change results in the amino acid substitution of isoleucine by serin (p.294Ile > Ser) and is classified as a polymorphism by MutationTaster or benign by Polyphen. The substitution p.294Ile > Ser is localized in the first PEST domain of PLK4 near the Degron motif with its serine and threonine residues (Fig. 2b), a peptide sequence hypothesized to target proteins for degradation (Rogers et al. 1986). PLK4 autophosphorylates the serine and threonine residues in the PEST domain to enhance its own degradation (Holland et al. 2010). In contrast to isoleucine serine can be phosphorylated. It is, therefore, plausible to assume that the mutation p.294Ile > Ser results in a further phosphorylatable site which could increase autodegradation of PLK4.

Thus, all affected individuals are compound heterozygous for the novel *PLK4* variant (c.881 T > G) and the deletion covering PLK4 (Fig. 2). The four affected adult patients of generation III reported here have a remarkably mild clinical phenotype compared to individuals with previously described PLK4 mutations. The latter show more severe microcephaly (up to  – 15 SD) and short stature (up to  – 8 SD), whereas two of our patients exhibit only mild short stature (III.3, III.4), two were in the lower normal range. Furthermore, our patients have only moderate Intellectual disability as all have attended a school for mentally handicapped. In particular their verbal skills are noteworthy, since all were able to speak in sentences, while the patients described in the literature have no speech or can speak only single words. Most reported patients have variable ophthalmological anomalies including retinopathy, microphthalmia, and optic nerve hypoplasia. A detailed ophthalmological examination could not be performed on our patients.

It is quite remarkable, that the grandmother, II.1, is homozygous for the variant c.881G but unaffected having a head circumference at 56.5 cm in the normal range. Thus, the pathogenic nature of the missense variant obviously becomes overt only in hemizygous individuals, when combined with the deletion of *PLK4* on the other chromosome. This suggests that the impairment of PLK4 function by mutation p.294Ile > Ser is without obvious phenotypic consequences in the homozygous state; however, compound heterozygote carriers of the deletion together with p.294Ile > Ser are at risk to be affected with microcephaly. That also explains the relatively mild clinical manifestations in the PLK4 patients reported here.

So far, our results of comprehensive genetic analyses using aCGH, WES, Sanger sequencing, and STR mapping are conclusive. However, that does not explain that genome wide SNP linkage analysis does not include *PLK4* in the compound heterozygote region in generation III. This prompted us to reconstruct the region around *PLK4* with SNP markers in the unaffected grandmother, II.1 and her offspring. In II.1 the homozygous *PLK4* variant c.811G is embedded in a 1.83 Mb homozygous region which is, however, localized on two different haplotypes (G1 and G2) (Fig. 3, Suppl. Figure 3). Consequently, II.1 inherits the variant c.881G to all offspring. The two affected individuals, III.2 and III.3, and their healthy brother, III.5 have the same maternal haplotype (G2). The affected proband III.4 carries the other maternal G allele (G1) and III.1 has a double crossover resulting in a switch from G2 to G1 proximal of PLK4 and back to G2 distal of PLK4 (Suppl. Figure 3). Thus, two affected carry the G1 und two affected the G2 haplotype around *PLK4* (Fig. 3). This explains also why this candidate region could not be detected by automated linkage analysis.

The sequence of 1.83 Mb around the *PLK4* locus is homozygous for all 84 SNPs on both haplotypes of II.1 (Fig. 3, Suppl. Figure 3). This could have resulted from a crossover in a heterozygote individual of a previous generation accompanied by gene conversion. Based on 158 WGS analyses of unrelated probands we found 3 cases with 0 to 2 heterozygote SNPs in this region (1.9%; range 0–50, median 24, Suppl. Table 2), also pointing to gene conversion. This is in line with the observation that such “complex crossovers” affect about 1% of all crossovers (Halldorsson et al. 2016; Halldorsson et al. 2019). Clearly, the 4q28 region is not a hotspot of recombination. However, the slightly higher crossover rate could be explained by its rather low GC content (Piovesan et al. 2019) and its late replication (Woodfine et al. 2004) both of which are characteristic for regions with higher crossover rates.

Thus, the clinical manifestations of the four affected family members of generation III. are due to either the mutated G1 or G2 haplotype inherited from II.1 (Fig. 3, Suppl. Figure 3) combined with the deletion of *PLK4*. The affected fetuses of generation IV received the G2 haplotype from their father and the deletion from their mother (Fig. 3). Altogether the deletion was inherited by 14 of 16 offspring, a significant deviation from expectation (*P* = 0.004, two-tailed binomial test). Four affected individuals of generation III inherited the deletion from their father (II.2), while the four affected fetuses of generation IV inherited it from their mother (III.6). In total, III.6 transmitted the deletion to seven of her eight children (Figs. 1, 3).

Interestingly, if the numbers of affected and unaffected individuals of the other families, published with *PLK4* mutations, are included, there is a significant overrepresentation of affected probands (Table 2; *P* = 0.002). Thus, the transmission ratio distortion is not confined to the *PLK4* deletion but also valid for the point mutations listed in Table 2. Without our family, the * p* value is 0,059. This is no longer significant, but in our impression still relevant, (if all affected homozygotes of the other families are included the *p* value is 0.0002).

**Table 2:** Comparison of the numbers of affected ( – / – ) and unaffected (+ / + ; ±) offspring in families with *PLK4* mutations

Family	Mutation	+ / + & ±	– / –	
Martin et al. 2014				Corr
- Pakistan	p.Arg936Serfs*1	10	7	6
- Iran	p.Phe433Leufs*6	–	1	0
- Madagaskar	p.Phe433Leufs*6	–	1	0
Shaheen et al. 2014	p.Phe433Leufs*6	2	3	2
Tsutsumi et al. 2016	p.M148V/p.C779Y	–	1	0
Dincer et al. 2017	p.Asp11Profs*14	2	3	2
Martin-Rivada et al. 2020	p.Phe433Leufs*6	2	2	1
This report				
- Generation IV	p.Ile294Ser/ Del	4	4	4
- Generation III*	p.Ile294Ser/ Del	1	4	3
	N observed	21	26	18
Two-tailed *P* = 0.002	N expected	29.25		9.75

Note: To calculate the expected number of affected individuals for statistical comparison (chi^2^ test), the index case from each family was omitted (corr.). The expected numbers are based on autosomal recessive inheritance with a 3: 1 segregation ratio, ^#^except for generation III with a 1: 1 ratio due to homozygosity of II.1 of the mutated allele

Examples of transmission ratio distortion (TRD) are rare because of a strong selection pressure for equal segregation of alleles (Nadeau 2017). TRD has been extensively studied in animals, has also been documented in human offspring of carriers with specific structural chromosome aberrations (Honeywell et al. 2012) and might also apply to specific loci in man (Meyer et al. 2012). Just recently Stabile et al (2020) described a preferential transmission of a mutation of the X-linked gene *ATRX* to the offspring. The most favoured mechanisms underlying TRD are meiotic drive (preferential transmission of a particular allele at meiosis), gametic competition (differential success of gametes in achieving fertilization), and embryo lethality (postzygotic selection against particular genotypes) (Huang et al. 2013).

In general, meiotic drive or gametic competition are restricted to one sex (Nadeau 2017) and are rather unlikely in the present family, since both spermatogenesis and oogenesis are involved in the preferential transmission of the *PLK4* deletion. Moreover, there is no evidence for spontaneous embryo lethality. Thus, the mechanisms discussed so far cannot explain the specific situation in this family.

It is well known that aneuploidy due to maternal meiotic non-disjunction can affect more than 50% of oocytes from women ≥ 40 years (MC 2019). Based on the analysis of human disaggregated cleavage-stage embryos, it is also evident that chromosomal errors are common during post-fertilization, leading to aneuploid mosaicism, which could rise to 95% in blastocysts (Starostik et al. 2020). In combination with time lapse imaging of developing embryos in vitro it could be shown that the incidence of abnormal mitoses with monopolar or multipolar spindles in the first three cleavage divisions affects about 25% of all embryos (Hlinka et al. 2012; Athayde Wirka et al. 2014; Kalatova et al. 2015; Zhan et al. 2016; Ottolini et al. 2017). Thus, it is realistic to assume that aneuploidy is a natural occurrence in early human embryos (Lee and Kiessling 2017; McCoy 2017).

In the present context it is relevant that early mitotic divisions are controlled both by maternal gene products, such as the key centrosomal proteins, including PLK4 (Alvarez Sedo et al. 2011) and by paternal elements provided by the sperm, such as the centriole (Palermo et al.^1994^; Sathananthan et al. 1996), for which PLK4 plays a key role in biogenesis (Nigg and Holland, 2018; Avidor-Reiss and Fishman 2019). The number of centrioles is a tightly controlled process to ensure a bipolar spindle and maintain genomic integrity (Ohta et al. 2018). While increased expression of *PLK4* is associated with centriole overduplication (Ganem et al. 2009), reduced expression leads to centriole loss (Bettencourt-Dias et al. 2011; Bettencourt-Dias et al. 2005).

Interestingly, a significant association has been reported between early mitotic aneuploidies and specific maternal haplotypes on chromosome 4 at q28.1 to q28.2, including *PLK4* (McCoy et al. 2015a, b; Zhang et al. 2017). One haplotype is associated with tripolar mitotic spindles leading to “chaotic mosaic aneuploidy” in cleavage-stage embryos (McCoy et al. 2017, 2018). In normally fertilized zygotes these tripolar spindles are the key mechanism contributing to the low rates of blastocyst formation. The other haplotype, with SNPs within the coding sequence of *PLK4,* in the kinase domain (rs3811740) and in the crypto Polo-box domain (rs17012739), is associated with less aneuploidy (McCoy et al. 2015a, b, 2018; Zhang et al. 2017). In our family all affected members are hemizygous for this haplotype (Suppl. Table 3).

Based on these observations it is tempting to speculate that the high rate of multipolar spindles during early mitotic divisions is due to the normally high expression of *PLK4*, resulting in reproductive failure. If the expression is reduced by deletion or mutation of the maternal or paternal *PLK4* allele, this could result in more bipolar spindles, leading to more viable diploid embryos. Consequently, the deleted *PLK4* allele is preferentially transmitted.

From an evolutionary point of view, it seems a paradox that the extremely high rate of meiotic and early mitotic aneuploidies, accompanied by reproductive failure, should represent a selective advantage. In fact, this is an adaptive mechanism to extend the interbirth interval from 9 months to 3–4 years, resulting in better overall survival rates. This mostly unnoticed failure reduces maternal costs which has been of particular importance in early human evolution (Lubinsky 2018; Valeggia and Ellison 2009). So far, there is no convincing evidence that *PLK4* mutations could exert a heterozygote advantage. However, one cannot exclude that *PLK4* heterozygotes could have a lower tumor risk at reproductive age, since PLK4 inhibitors do indeed suppress tumor growth in vitro and in vivo (Zhao and Wang 2019; Raab et al. 2021).

Unfortunately, the effect of PLK4 “normalization” cannot be tested in the mouse model, because mouse sperm and zygotes appear to lack centrioles (Avidor-Reiss and Fisherman 2019; Avidor Reis et al. 2020) and the rate of aneuploidies in mouse embryos is more than an order of magnitude lower than in man (Bond and Chandley 1983). In the future, the in vitro generation of human primordial germ cells from pluripotent stem cells may allow the investigation of the mechanisms of early human embryonic cell divisions, including the role of PLK4 (Jung et al. 2017; Hayashi et al. 2018).

## Supplementary Information

Below is the link to the electronic supplementary material.

Supplementary file1 (PDF 642 KB)

## Data Availability

The data that support the findings of this study are available in this article. The additional data sets generated during the current study (i.e., 10 K, 250 K SNP arrays, STR analysis, Sanger Sequencing) are available from the corresponding author on reasonable request.
