# Supplementary material for: Transmission ratio distortion of mutations in the master regulator of centriole biogenesis PLK4
Source: Hum Genet. 2022 May 10;141(11):1785–94. doi: 10.1007/s00439-022-02461-w (PMC9556372; doi:10.1007/s00439-022-02461-w)
Supplement: Supplementary file 1 — Supplementary file1 (PDF 642 KB) [file 439_2022_2461_MOESM1_ESM.pdf]

S2

Supplementary Table S2. Number of [illegible]

[illegible]
